# Prediction, Suppression of Visual Response, and Modulation of Visual Perception: Insights From Visual Evoked Potentials and Representational Momentum

**DOI:** 10.3389/fnhum.2021.730962

**Published:** 2021-08-25

**Authors:** Motohiro Kimura

**Affiliations:** Department of Information Technology and Human Factors, National Institute of Advanced Industrial Science and Technology (AIST), Tsukuba, Japan

**Keywords:** visual evoked potentials (VEPs), representational momentum, visual perception, prediction suppression, central P2, individual difference

## Abstract

When a visual object changes its position along with certain sequential regularities, the visual system rapidly and automatically forms a prediction regarding the future position of the object based on the regularities. Such prediction can drastically alter visual perception. A phenomenon called representational momentum (RM: a predictive displacement of the perceived final position of a visual object along its recent regular pattern) has provided extensive evidence for the predictive modulation of visual perception. The purpose of the present study was to identify neural effects that could explain individual differences in the strength of the predictive modulation of visual perception as measured by RM. For this purpose, in two experiments with a conventional RM paradigm where a bar was discretely presented in a regular rotation manner (with a step of 18° in Experiment 1 and a step of 20° in Experiment 2), visual evoked potentials (VEPs) in response to the regularly rotated bar were measured, and correlations between the magnitudes of RM and VEPs were examined. The results showed that the magnitudes of RM and central P2 were negatively correlated, consistently in both experiments; participants who showed a smaller central P2 tended to exhibit greater RM. Together with a previous proposal that central P2 would represent delayed reactivation of lower visual areas around the striate and prestriate cortices via reentrant feedback projections from higher areas, the present results suggest that greater suppression of delayed reactivation of lower visual areas (as indicated by smaller central P2) may underlie stronger predictive modulation of visual perception (as indicated by greater RM).

## Introduction

Visual objects in the environment (e.g., a flying ball) dynamically change their positions. However, when an object’s image hits an observer’s eyes, the observer cannot perceive the image instantaneously; it takes about a tenth of a second after the image hits the eyes. Therefore, by the time the observer has perceived the object at a certain position, its actual position has already changed. Despite this fundamental problem, an observer can effortlessly interact with such objects in real time (e.g., by catching a flying ball). A possible solution to the problem of how the visual system can bridge the gap between perception and action is to form a prediction about the future position of the object, based on sequential regularities in the recent past (i.e., recent trajectory of the ball) (Mackay, 1958; Freyd, 1992; Nijhawan, 1994; Hubbard, 1995, 2005).

A phenomenon known as representational momentum (RM: Freyd and Finke, 1984, 1985) provides strong evidence for the existence of such prediction based on sequential regularities in the recent past and demonstrates that visual perception can indeed be strongly modulated by the prediction. RM denotes predictive displacement of the perceived final position of a changing object. In a conventional RM paradigm developed by Freyd and Finke (1984, 1985), participants observe a stimulus sequence where a bar is discretely presented in a regular rotation manner (denoted “inducing stimuli”: e.g., 10°/30°/50°). Participants are required to compare the orientation of the final inducing stimulus (i.e., 50°) to that of a subsequent bar (denoted “probe”). It has been shown that participants report “same” with higher probability when the probe is slightly shifted forward along the regular direction of rotation (e.g., 52°) than when it is truly the same (50°) or shifted backward (e.g., 48°) (Freyd and Finke, 1985). RM is thought to reflect predictive displacement of the sensory representation of an object along its recent change pattern (Freyd, 1992; Hubbard, 1995, 2005). RM can be observed based on sequential regularities in position or orientation but also in other visual features (Kelly and Freyd, 1987; Hayes and Freyd, 2002) and sequential regularities in auditory features (Freyd et al., 1990), suggesting that the predictive displacement of sensory representation would be a general phenomenon across visual features and sensory modalities. Also, RM can occur without the observer paying much attention to the object (Hayes and Freyd, 2002; for related findings, see Finke and Freyd, 1985), suggesting that the predictive displacement of sensory representation can occur in an automatic and obligatory manner.

**FIGURE 1:**
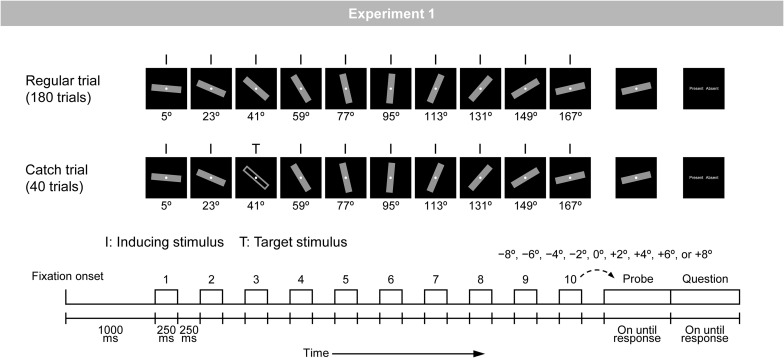
Schematic illustration of the regular and catch trials in Experiment 1. A bar was rotated regularly with a step of 18°.

**FIGURE 2:**
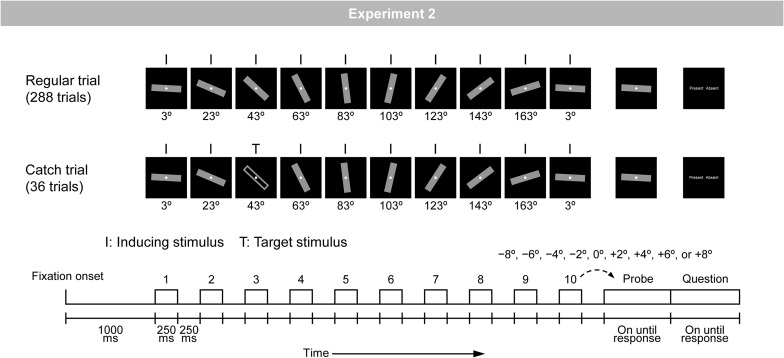
Schematic illustration of the regular and catch trials in Experiment 2. A bar was rotated regularly with a step of 20°.

Representational momentum is a robust phenomenon that is stably observed across participants (Freyd and Finke, 1985). However, there seem to be large individual differences in the magnitude of RM (Finke et al., 1986; Verfaillie and d’Ydewalle, 1991), which leads to the assumption that there may be large individual differences in the strength of the predictive modulation of visual perception. The purpose of the present study was to identify neural effects that could explain individual differences in the strength of predictive modulation of visual perception as measured by RM. For this purpose, the present study measured visual evoked potentials (VEPs) with a conventional RM paradigm (Freyd and Finke, 1984, 1985). In two experiments, a bar was discretely presented in a regular rotation manner (i.e., inducing stimuli); with a step of 18° in Experiment 1 (Figure 1) and 20° in Experiment 2 (Figure 2). Participants were required to compare the orientation of the final (i.e., tenth) inducing stimulus to that of a subsequent probe. VEPs in response to inducing stimuli were measured, and correlations were examined between the magnitudes of RM and VEPs: (1) occipito-temporal P1 at around 110 ms, (2) frontal N1 at around 140 ms, (3) occipito-temporal N1 at around 170 ms, and (4) central P2 at around 200 ms after stimulus onset (Clark et al., 1995; Di Russo et al., 2002; Capilla et al., 2016).^1^

No previous study has examined the relationship between the magnitudes of RM and VEPs in response to inducing stimuli. However, based on a previous VEP finding on automatic prediction based on sequential regularities (Kimura and Takeda, 2015), the neural effect that is most likely to correlate with RM is central P2. To identify neural effects that specifically emerge when the current position of an object successfully matches the predicted position of the object based on sequential regularities, Kimura and Takeda (2015) compared VEPs elicited by bars that were discretely presented in a regular rotation manner (e.g., 10°/30°/50°/70°/90°…, where the upcoming orientation of the bar could be predicted, as in the RM paradigm) to VEPs elicited by the same bars that were discretely presented in a random manner (e.g., 70°/10°/30°/90°/50°…, where a prediction of the upcoming orientation of the bar could not be formed). It was found that central P2 at around 200 ms after stimulus onset was selectively suppressed when the upcoming orientation could be predicted compared to when a prediction could not be formed. Contrary to central P2, no difference in this comparison was found for occipito-temporal P1 at around 110 ms, frontal N1 at around 140 ms, and occipito-temporal N1 at around 170 ms; instead, occipito-temporal P1 and N1 (but not frontal N1) were found to be suppressed only when bars were presented in a repetitive manner (e.g., 10°/10°/10°/10°/10°…) compared to when bars were presented in a random manner (e.g., 70°/10°/30°/90°/50°…), suggesting that these P1 and N1 effects represent repetition suppression attributable to stimulus-specific adaptation or neural refractoriness rather than prediction suppression (cf. Todorovic and de Lange, 2012). Therefore, at least when a regularly rotating bar was used as stimuli, the suppression of central P2 is thought to be a unique neural effect that could emerge when the current position of a visual object successfully matches the predicted position of the object based on sequential regularities.

The neural sources of central P2 at around 200 ms were previously localized in lower visual areas around the striate and prestriate cortices (Capilla et al., 2016); although the neural sources of P2 are still less well understood compared to those of other VEPs, this finding appears to be consistent with a non-human neuroimaging finding suggesting the involvement of lower visual areas (i.e., monkey V2) in the P2 homolog (Metha et al., 2000). The involvement of lower visual areas in P2 is interesting, since the neural sources of temporally earlier VEPs such as P1 at around 110 ms were localized in higher visual areas including the dorsal and ventral extrastriate cortices (Clark et al., 1995; Di Russo et al., 2002). To explain this paradox, P2 has proposed to be a sign of delayed reactivation of lower visual areas via reentrant feedback projections from higher areas (Di Russo et al., 2003, 2008). Based on these previous findings, prediction suppression of central P2 (Kimura and Takeda, 2015) is best assumed to represent reduced delayed reactivation of lower visual areas around the striate and prestriate cortices. This assumption is consistent with human neuroimaging findings that automatic prediction based on sequential regularities resulted in suppressed activation in lower visual areas including the striate cortex, whereas activation in higher visual areas including the dorsal extrastriate cortex was not affected (Alink et al., 2010) and non-human neuroimaging findings that automatic prediction based on sequential regularities resulted in markedly suppressed activation in lower visual areas (i.e., monkey V2) rather than higher visual areas (Vergnieux and Vogels, 2020; see also Kaposvari et al., 2018).

Taken together, the present study expected that participants who exhibited greater RM may show smaller central P2 in response to inducing stimuli; in other words, the magnitudes of RM and central P2 would show a negative correlation.

## Experiment 1

The experiment reported here was conducted with multiple purposes, and included trials that were not related to the present purpose (i.e., irregular trials; see Materials and Methods). Data in the irregular trials have already been reported in another paper (Kimura, 2018). Data reported in this paper have not been reported elsewhere.

### Materials and Methods

#### Participants

Thirty-five healthy adults (32 males, 3 females; mean age 22.5 years; age range 19–32 years) participated in this experiment. All participants had normal or corrected-to-normal vision. Thirty-three participants were right-handed and two were left-handed. Written informed consent was obtained from each participant after the nature of the study had been explained. The experiment was approved by the Safety and Ethics committee of the National Institute of Advanced Industrial Science and Technology (AIST).

#### Stimuli and Procedure

The experiment was controlled by MATLAB (MathWorks) on Mac OSX with the Psychophysics Toolbox (Brainard, 1997; Pelli, 1997). All visual stimuli were presented on a 17-inch cathode ray tube display (Sony, Trinitron Multiscan G220) at a viewing distance of about 57 cm.

The experiment consisted of three types of trials (i.e., regular, irregular, and catch trials). Figure 1 shows an illustration of the regular and catch trials; the irregular trials are not related to the present purpose and therefore are not illustrated in Figure 1. The regular trial was included to measure RM and the catch trial was included to ensure that participants kept observing the stimulus sequence. Each trial began with the onset of a gray fixation circle (42.3 cd/m^2^; diameter of 0.3°), which was continuously visible on the display. At 1000 ms after fixation onset, a stimulus sequence consisting of 10 presentations of a bar appeared. In the regular trial, a gray-filled bar (9.2 cd/m^2^; width of 0.9° × height of 5.7°) was rotated regularly with a step of 18° (i.e., inducing stimuli). In the catch trial, a gray-filled bar was rotated regularly, but at any of the 10 positions, it was replaced with a gray-unfilled bar (i.e., target stimuli). In all trials, each stimulus was presented for 250 ms and the inter-stimulus interval, where only the fixation circle was presented, was 250 ms. Note that 10 presentations of inducing stimuli would not necessarily be needed to obtain RM, given that three, four, or at most five presentations of inducing stimuli are common in RM studies. In the present study, 10 presentations were adopted to ensure that prediction had been fully stabilized by the time probe was presented.

This stimulus sequence was followed by a probe. The orientation of the probe was either the same as or slightly different than that of the final (i.e., tenth) inducing stimulus (i.e., −8°, −6°, −4°, −2°, 0°, +2°, +4°, +6°, or +8°). Here, the participants judged whether the orientations of the final inducing stimulus and the probe were the same or different, by pressing either the left or right response button. Mapping of same/different judgments and left/right buttons was fixed throughout the experiment for each participant and counterbalanced across participants. The probe was presented until the participant’s response.

The participant’s response was immediately followed by a question display consisting of the words “Present” and “Absent.” Here, the participants judged whether the target stimulus (i.e., a gray-unfilled bar presented only in the catch trial) was presented or not, by pressing either the left or right response button beside the words on the display. The side on which the words were presented was varied randomly across trials, with a constraint that two possible arrangements (i.e., “Present” on the left and “Absent” on the right, and vice versa) were equally presented within an experiment. The question display was presented until the participant’s response, which was immediately followed by a blank screen for 2000 ms.

The experiment included 180 regular trials and 40 catch trials, which were arranged in random order. In the 180 regular trials, 20 trial types, defined by the combination of 10 orientations of the first inducing stimulus (i.e., from 5° to 167° with a step of 18°) and two directions of regular rotation (i.e., clockwise and counterclockwise), were presented in nine trials each. In these 180 trials, nine angular differences between the final inducing stimulus and the probe (i.e., −8°, −6°, −4°, −2°, 0°, +2°, +4°, +6°, and +8°) were assigned with equal probabilities. Note that the 10 orientations of the first inducing stimulus were used to keep the physical attributes of inducing stimuli presented at each of the 10 positions in a stimulus sequence on average the same. That is, at each of 10 positions, 10 orientations were presented 18 times each.

In the 40 catch trials, the same 20 trial types, defined by the combination of 10 orientations of the first inducing stimulus (i.e., from 5° to 167° with a step of 18°) and two directions of regular rotation (i.e., clockwise and counterclockwise), were presented in two trials each. In these 40 trials, the target stimulus was presented at each of 10 positions with equal probability. The orientations of the final inducing stimulus and the probe were always the same.

The participants performed the task while seated in a chair in a sound-attenuated, dimly lit room. Before the start of the experiment, the participants were given instructions about the same/different judgment. They were instructed to judge whether the orientations of the tenth stimulus and a subsequent probe were the same or different, as accurately as possible. They were also instructed to count stimuli so that the tenth stimulus could be properly compared with the probe. The speed of their response was not stressed. Here, they were explicitly informed that the angular difference would be −8°, −6°, −4°, −2°, 0°, +2°, +4°, +6°, or +8°; this was intended to help the participants understand that the angular difference would be quite small. However, they were not informed about the ratio of “same” and “different” trials. Information regarding the nine angular differences might have led participants to expect the probability of each angular difference to be about 11%. However, such expectation is unlikely to significantly affect the magnitude of RM, although it may affect the overall probability of making a “same” response (Hubbard and Lange, 2010). Finally, it was emphasized that they should make a “same” response only when they believed that the orientations were exactly the same (Freyd and Finke, 1985).

Next, the participants were given instructions about the present/absent judgment. They were instructed to judge whether or not an unfilled stimulus was presented, as accurately as possible. The speed of their response was not stressed. Here, they were explicitly informed that the unfilled stimulus could appear at any of 10 positions in the stimulus sequence. However, they were not informed about the ratio of “present” and “absent” trials. It was emphasized that they should keep observing the stimulus sequence to perform this task adequately.

Finally, the participants were instructed to minimize any eye movements and blinks when the stimulus sequence was presented. After these instructions, the participants performed 20–40 practice trials, and then started the experiment.

#### Recordings

The electroencephalogram (EEG) was recorded with a digital amplifier (Nihon-Kohden, Neurofax EEG1200) and silver-silver chloride electrodes placed at 27 scalp sites (Fp1, Fp2, F7, F3, Fz, F4, F8, FCz, T7, C3, Cz, C4, T8, CPz, P7, P3, Pz, P4, P8, PO7, PO3, POz, PO4, PO8, O1, Oz, and O2 according to the extended International 10–20 System). All electrodes were referenced to the nose tip. To monitor blinks and eye movements, vertical and horizontal electrooculograms (EOGs) were also recorded with two electrodes above and below the right eye and two electrodes at the right and left outer canthi of the eyes, respectively. The ground electrode was attached to the forehead. The impedance of all electrodes was kept below 5 kΩ. The EEG and EOG signals were bandpass-filtered online at 0.016–300 Hz and digitized at a sampling rate of 1000 Hz.

The digitized signals were then analyzed by MATLAB (MathWorks) with EEGLAB toolbox (Delorme and Makeig, 2004) and ERPLAB Toolbox (Lopez-Calderon and Luck, 2014). The EEG and EOG signals were bandpass-filtered using a non-causal Butterworth infinite impulse response filter with half-amplitude cutoffs at 0.1 and 30 Hz and a roll-off of 12 dB/octave. The EEG and EOG signals time-locked to the onset of inducing stimuli were extracted. The extracted epochs were 600 ms (i.e., from −100 to 500 ms relative to the onset of inducing stimuli). An independent component analysis (Delorme and Makeig, 2004) was performed to remove artifacts derived from blinks and eye movements. The epochs were then baseline-corrected relative to the initial 100-ms interval (i.e., from −100 to 0 ms relative to the onset of inducing stimuli).

For each participant, the EEG signals in the regular trials were averaged for four categories: i.e., inducing stimuli (1) at the first position, (2) at the second, third, and fourth positions, (3) at the fifth, sixth, and seventh positions, and (4) at the eighth, ninth, and tenth positions. VEPs elicited by inducing stimuli at the first position were separately averaged, in consideration of their special morphologies reflecting initial-orienting reaction (Kenemans et al., 1989). VEPs elicited by inducing stimuli at the second–tenth positions were separated for three categories, to explore the time course of the correlation of RM and VEPs, while meeting ideal averaging numbers for VEPs (i.e., about 400 times, Luck, 2005). Note that the physical attributes of the inducing stimuli for these four position categories were on average kept the same. Epochs during which the signal change exceeded ± 80 μV on any of the EEG or EOG electrodes were excluded from averaging. As a result, the number of epochs averaged for the first, second–fourth, fifth–seventh, and eighth–tenth positions was, on average, 170.8 (*SD* = 10.2), 524.5 (20.9), 530.1 (14.7), and 532.8 (10.4), respectively.

#### Data Analysis

##### Magnitude of RM

For each participant, the percentages of “same” responses in the regular trials were calculated for nine position categories defined by the angular difference between the final inducing stimulus and the probe and its relation to the direction of regular rotation (i.e., backward 8°, backward 6°, backward 4°, backward 2°, same, forward 2°, forward 4°, forward 6°, and forward 8°). Next, for each participant, the magnitude of RM was estimated by a standard formula for calculating the mean position of a probe judged as “same” (Freyd and Jones, 1994; Hayes and Freyd, 2002; Munger and Minchew, 2002). In this calculation, each “same” response was weighted by the position of the probe, and the average of these weighted “same” responses was estimated to be the magnitude of RM.^2^ To confirm the occurrence of RM, the measured values were compared to zero with a one-tailed *t*-test; the statistical threshold was *p* < 0.05.

##### Target detection

For each participant, the percentage of “present” responses in the catch trials (i.e., hit rate) and those of “absent” responses in the regular trials (i.e., correct rejection rates) were calculated.

##### Magnitude of VEPs

For each participant, the magnitudes of VEPs elicited by inducing stimuli in the regular trials were estimated by calculating the mean amplitudes of the occipito-temporal P1, frontal N1, occipito-temporal N1, and central P2 for the four position categories (i.e., first, second–fourth, fifth–seventh, and eighth–tenth positions). The time windows of these VEPs for the second–fourth, fifth–seventh, and eighth–tenth positions were determined to be the 40-ms windows centered on the peaks in the grand-average VEPs in which the three position categories were collapsed; this procedure was chosen to avoid possible biases among the three position categories (Luck, 2014). As a result, the time windows were determined as follows: within the 90–130 ms time window at the PO8 electrode site for occipito-temporal P1, within the 118–158 ms time window at the Fz electrode site for frontal N1, within the 148–188 ms time window at the PO8 electrode site for occipito-temporal N1, and within the 178–218 ms time window at the Cz electrode site for central P2 (Table 1). The time windows for the first position were separately determined as the 40-ms windows centered on the peaks in the grand-average VEPs for the first position. As a result, the time windows were determined as follows: within the 94–134 ms time window at the PO8 electrode site for occipito-temporal P1, within the 119–159 ms time window at the Fz electrode site for frontal N1, within the 150–190 ms time window at the PO8 electrode site for occipito-temporal N1, and within the 212–252 ms time window at the Cz electrode site for central P2 (Table 1).

**TABLE 1:** Time windows for calculating mean amplitudes of VEPs in Experiment 1.

	Position 1	Positions 2–4, 5–7, and 8–10
Occipito-temporal P1	94–134 ms (PO8)	90–130 ms (PO8)
Frontal N1	119–159 ms (Fz)	118–158 ms (Fz)
Occipito-temporal N1	150–190 ms (PO8)	148–188 ms (PO8)
Central P2	212–252 ms (Cz)	178–218 ms (Cz)

##### Correlations between RM and VEPs

The correlation between the magnitudes of RM and VEPs (i.e., occipito-temporal P1, frontal N1, occipito-temporal N1, and central P2 for the four position categories) were assessed by Spearman’s correlation analyses (two-tailed); the statistical threshold was *p* < 0.05. Spearman’s correlation analysis was chosen here, since the relationship between RM and VEPs was assumed to be not necessarily linear.

### Results

**FIGURE 3:**
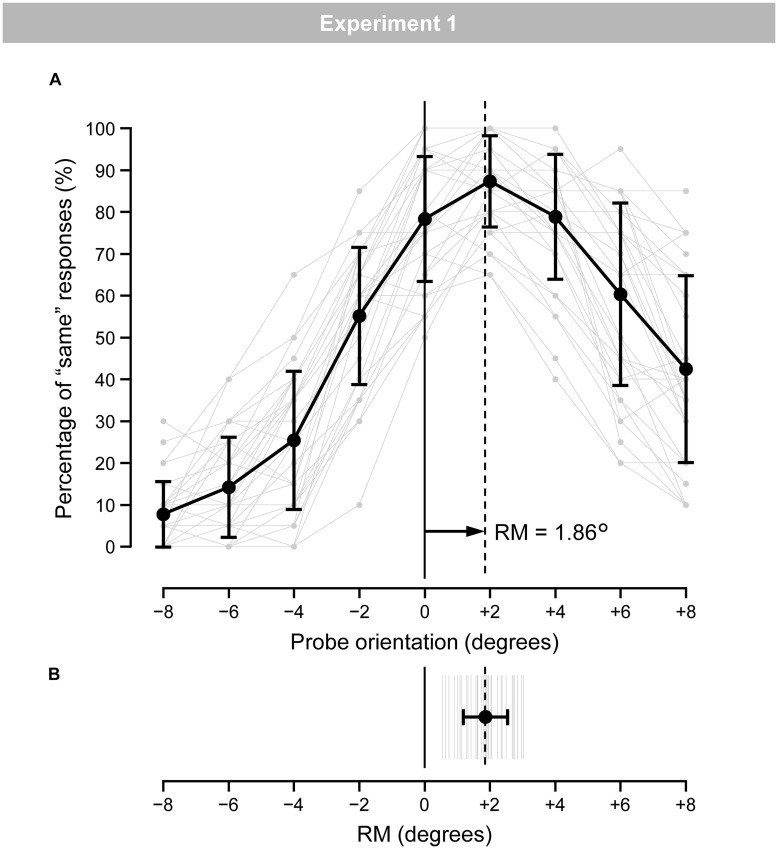
**(A)** Mean (black) and individual (gray) percentages of “same” responses for the nine probe-orientation categories in the regular trials. Error bars indicate *SD*. **(B)** Mean (black) and individual (gray) magnitudes of RM. Error bars indicate *SD*.

Figure 3A shows the mean (black line) and individual (gray lines) percentages of “same” responses. Figure 3B shows the mean (black line) and individual (gray lines) magnitudes of RM. The individual magnitudes of RM ranged from 0.55° to 3.02°. The mean magnitude of RM was 1.86° (*SD* = 0.68). A one-tailed *t*-test revealed a significant occurrence of RM [*t*(34) = 16.31, *p* < 0.001, *d* = 2.76].

The mean hit rate in the catch trial was 95.1% (*SD* = 8.4). The mean correct rejection rate in the regular trial was 98.9% (*SD* = 1.1).

**FIGURE 4:**
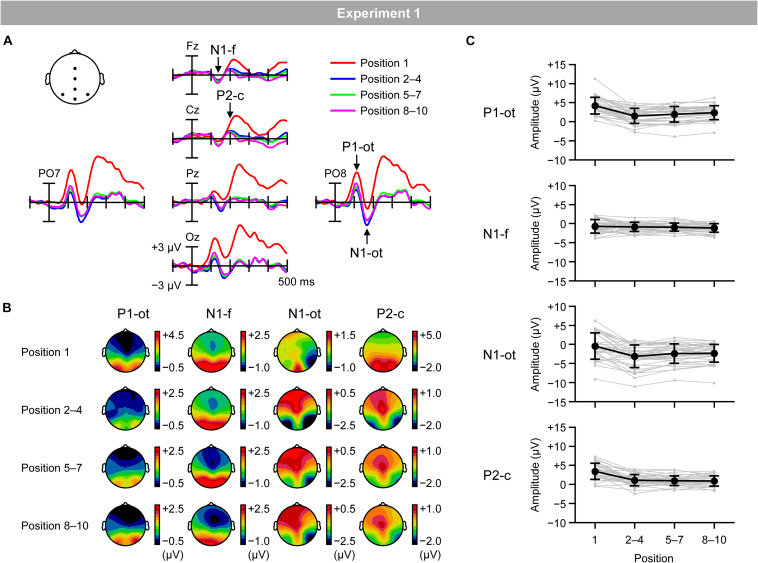
**(A)** VEPs elicited by inducing stimuli presented at the first position (red lines), second, third, and fourth positions (blue lines), fifth, sixth, and seventh positions (green lines), and eighth, ninth, and tenth positions (purple lines) in the regular trials. **(B)** Topographical maps of VEPs. **(C)** Mean (black) and individual (gray) magnitudes of VEPs. P1-ot: occipito-temporal P1, N1-f: frontal N1, N1-ot: occipito-temporal N1, P2-c: central P2. Error bars indicate *SD*.

Figure 4A shows VEPs elicited by the inducing stimuli in the regular trials for the first (red lines), second–fourth (blue lines), fifth–seventh (green lines), and eighth–tenth positions (purple lines). Figure 4B shows topographical maps of VEPs within the time windows listed in Table 1. Typical waveforms consisting of occipito-temporal P1, frontal N1, occipito-temporal N1, and central P2 were observed. Figure 4C shows the mean (black lines) and individual (gray lines) magnitudes of these VEPs, calculated as the mean amplitude according to the time windows and electrodes sites listed in Table 1.

**FIGURE 5:**
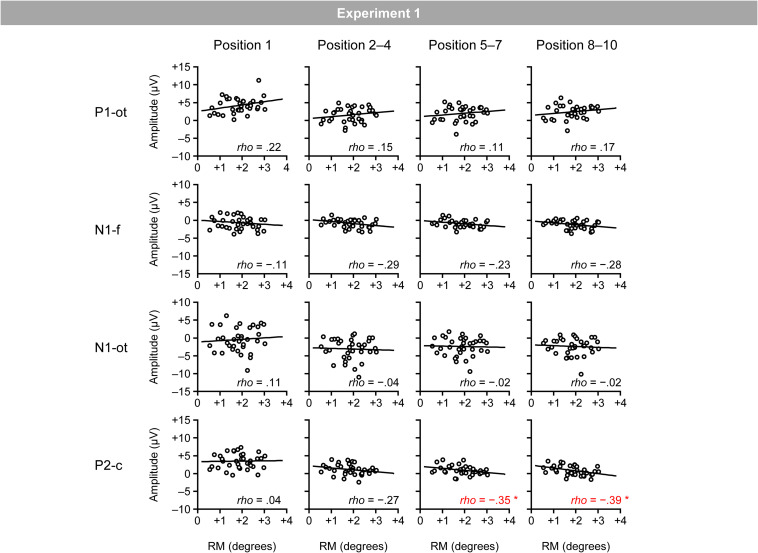
Scatter plots of the relationships between the magnitudes of RM and VEPs. P1-ot: occipito-temporal P1, N1-f: frontal N1, N1-ot: occipito-temporal N1, P2-c: central P2. The linear regression fits to the data are shown. *Indicates *p* < 0.05 by Spearman’s correlation analysis (two-tailed).

Figure 5 shows the relationship between the magnitudes of RM and VEPs (i.e., occipito-temporal P1, frontal N1, occipito-temporal N1, and central P2 for the four position categories). Spearman’s correlation analysis (two-tailed) revealed significant negative correlations between the magnitudes of RM and central P2 for the fifth–seventh (ρ = −0.35; *p* < 0.05) and eighth–tenth positions (ρ = −0.39; *p* < 0.05).^3^

### Discussion

The results regarding the same/different judgment showed that RM robustly occurred in the regular trials. This is highly consistent with previous RM findings (Freyd and Finke, 1984, 1985). The results regarding target detection showed that the hit rates in the catch trial as well as the correct rejection rates in the regular trial were high, ensuring that the participants kept observing the stimulus sequence. The results regarding VEPs showed that inducing stimuli elicited occipito-temporal P1, frontal N1, occipito-temporal N1, and central P2 that were comparable to those obtained with regularly rotated bars (Kimura and Takeda, 2015). For the correlation between RM and VEPs, the magnitude of RM was negatively correlated with the magnitude of central P2; participants who showed a smaller P2 tended to exhibit greater RM. This seems to be consistent with a previous finding that the suppression of central P2 would be a neural effect that would specifically emerge when the current and predicted positions of an object successfully matched (Kimura and Takeda, 2015). In contrast to central P2, the magnitude of RM was not correlated with the magnitude of occipito-temporal P1, frontal N1, and occipito-temporal N1. Given a previous finding that these VEPs were not sensitive to successful matching between the current and predicted positions of a visual object (Kimura and Takeda, 2015), the null correlation seems to be reasonable.

## Experiment 2

To test the replicability and robustness of the negative correlation between the magnitudes of RM and central P2, the same analyses were performed on data obtained in another experiment where a bar was regularly rotated with a different angular step (i.e., 20°; cf. 18° in Experiment 1). Similar to Experiment 1, the experiment reported here was conducted with multiple purposes, and included trials that were not related to the present purpose (i.e., irregular trials; see Materials and Methods). Data in the irregular condition will be reported elsewhere. Data reported in this paper have not been reported elsewhere.

### Materials and Methods

#### Participants

Thirty-seven healthy adults (26 males, 11 females; mean age 23.3 years; age range 20–33 years) participated in this experiment; three participants had also participated in Experiment 1. All participants had normal or corrected-to-normal vision. Thirty-six participants were right-handed and one was left-handed. Written informed consent was obtained from each participant after the nature of the study had been explained. The experiment was approved by the Safety and Ethics committee of the National Institute of Advanced Industrial Science and Technology (AIST).

#### Stimuli and Procedure

The stimuli and procedure were the same as those in Experiment 1, except for the following points. The experiment was comprised of three types of trials (i.e., regular, irregular, and catch trials). Figure 2 shows an illustration of the regular and catch trials. In the regular trial, a gray-filled bar was rotated regularly with a step of 20° (i.e., inducing stimuli). In the catch trial, a gray-filled bar was rotated regularly, but at any of the 10 positions, it was replaced by a gray-unfilled bar (i.e., target stimuli).

The experiment included 288 regular trials and 36 catch trials, which were arranged in random order. The direction of regular rotation was fixed throughout the experiment for each participant and counterbalanced across the participants; for half of the participants (i.e., 18 participants), the direction of regular rotation was clockwise, and for the other half of the participants (i.e., 19 participants), the direction of regular rotation was counterclockwise.

In the 288 regular trials, 36 trial types that were defined by 36 orientations of the first inducing stimulus (i.e., from 3° to 178° with a step of 5°) were presented in eight trials each. In these 288 trials, nine angular differences between the final inducing stimulus and the probe (i.e., −8°, −6°, −4°, −2°, 0°, +2°, +4°, +6°, and +8°) were assigned with equal probabilities. Note that the 36 orientations of the first stimulus were used to keep the physical attributes of inducing stimuli presented at each of the 10 positions in a stimulus sequence on average the same. Thus, at each of the 10 positions, 36 orientations were presented eight times each.

In the 36 catch trials, the same 36 trial types that were defined by 36 orientations of the first stimulus (i.e., from 3° to 178° with a step of 5°) were presented in one trial each. In these 36 trials, the target stimulus was presented at each of 10 positions with almost equal probability.

#### Recordings

The recording parameters were the same as those in Experiment 1. As a result, the number of epochs averaged for the first, second–fourth, fifth–seventh, and eighth–tenth positions was, on average, 280.4 (*SD* = 12.1), 851.2 (29.8), 856.2 (21.1), and 857.8 (10.7), respectively.

#### Data Analysis

##### Magnitude of RM

The data analysis was the same as that in Experiment 1.

##### Target detection

The data analysis was the same as that in Experiment 1.

##### Magnitude of VEPs

The data analysis was the same as that in Experiment 1, except for the time windows for calculating the mean amplitudes of VEPs. The time windows of VEPs for the second–fourth, fifth–seventh, and eighth–tenth positions were determined as follows: within the 89–129 ms time window at the PO8 electrode site for occipito-temporal P1, within the 111–151 ms time window at the Fz electrode site for frontal N1, within the 146–186 ms time window at the PO8 electrode site for occipito-temporal N1, and within the 174–214 ms time window at the Cz electrode site for central P2 (Table 2). The time windows of VEPs for the first position were determined as follows: within the 100–140 ms time window at the PO8 electrode site for occipito-temporal P1, within the 114–154 ms time window at the Fz electrode site for frontal N1, within the 151–191 ms time window at the PO8 electrode site for occipito-temporal N1, and within the 200–240 ms time window at the Cz electrode site for central P2 (Table 2).

**TABLE 2:** Time windows for calculating mean amplitudes of VEPs in Experiment 2.

	Position 1	Positions 2–4, 5–7, and 8–10
Occipito-temporal P1	100–140 ms (PO8)	89–129 ms (PO8)
Frontal N1	114–159 ms (Fz)	111–151 ms (Fz)
Occipito-temporal N1	151–191 ms (PO8)	146–186 ms (PO8)
Central P2	200–240 ms (Cz)	174–214 ms (Cz)

##### Correlations between RM and VEPs

The analysis was the same as that in Experiment 1.

### Results

**FIGURE 6:**
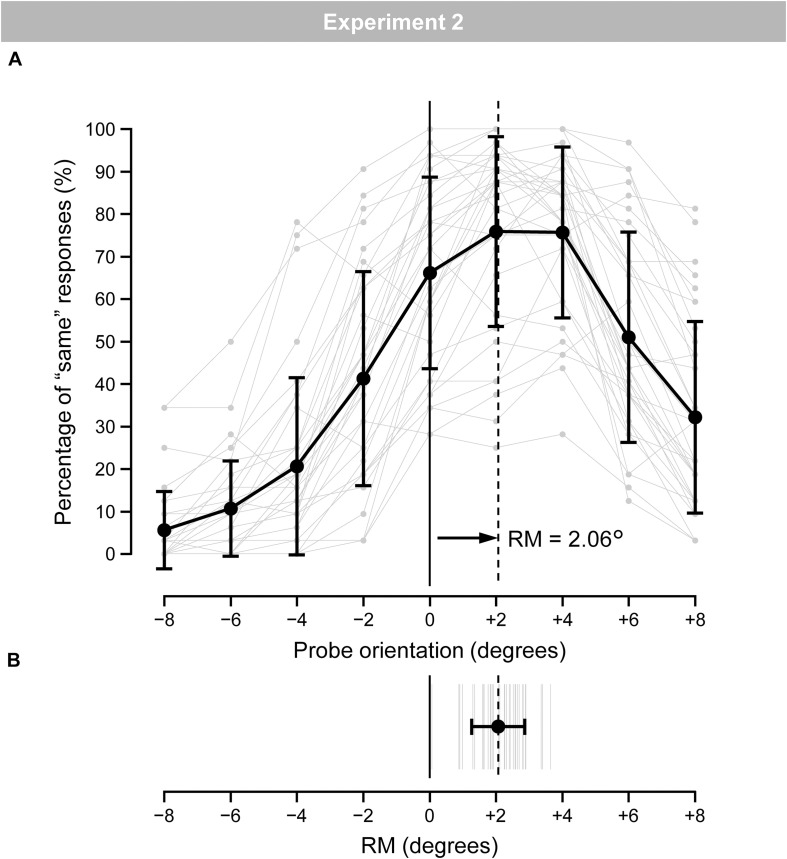
**(A)** Mean (black) and individual (gray) percentages of “same” responses for the nine probe-orientation categories in the regular trials. Error bars indicate *SD*. **(B)** Mean (black) and individual (gray) magnitudes of RM. Error bars indicate *SD*.

Figure 6A shows the mean (black line) and individual (gray lines) percentages of “same” responses. Figure 6B shows the mean (black line) and individual (gray lines) magnitudes of RM. The individual magnitudes of RM ranged from 0.07° to 3.65°. The mean magnitude of RM was 2.06° (*SD* = 0.80). A one-tailed *t*-test revealed a significant occurrence of RM [*t*(36) = 15.73, *p* < 0.001, *d* = 2.59].

The mean hit rate in the catch trial was 94.7% (*SD* = 6.2). The mean correct rejection rate in the regular trial was 98.6% (*SD* = 1.1).

**FIGURE 7:**
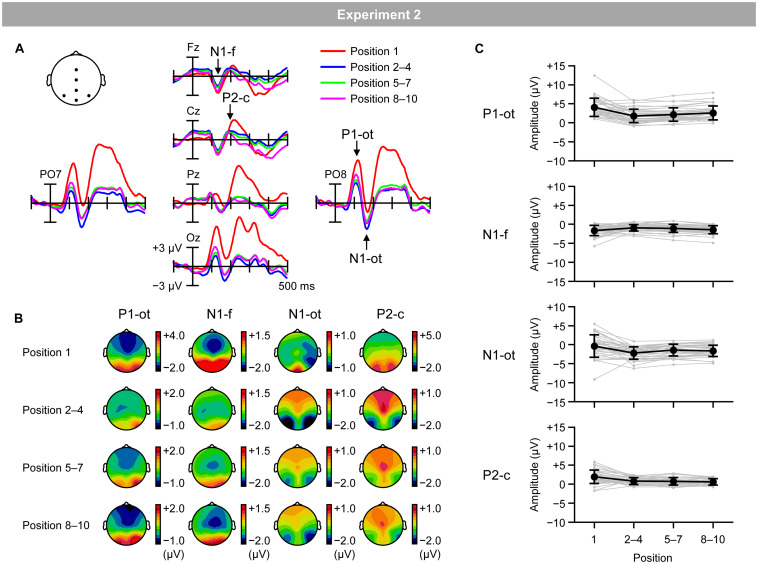
**(A)** VEPs elicited by inducing stimuli presented at the first position (red lines), second, third, and fourth positions (blue lines), fifth, sixth, and seventh positions (green lines), and eighth, ninth, and tenth positions (purple lines) in the regular trials. **(B)** Topographical maps of VEPs. **(C)** Mean (black) and individual (gray) magnitudes of VEPs. P1-ot: occipito-temporal P1, N1-f: frontal N1, N1-ot: occipito-temporal N1, P2-c: central P2. Error bars indicate *SD*.

Figure 7A shows VEPs elicited by the inducing stimuli in the regular trials for the first (red lines), second–fourth (blue lines), fifth–seventh (green lines), and eighth–tenth positions (purple lines). Figure 7B shows topographical maps of VEPs within the time windows listed in Table 2. Figure 7C shows the mean (black lines) and individual (gray lines) magnitudes of VEPs, calculated as the mean amplitude according to the time windows and electrodes sites listed in Table 2.

**FIGURE 8:**
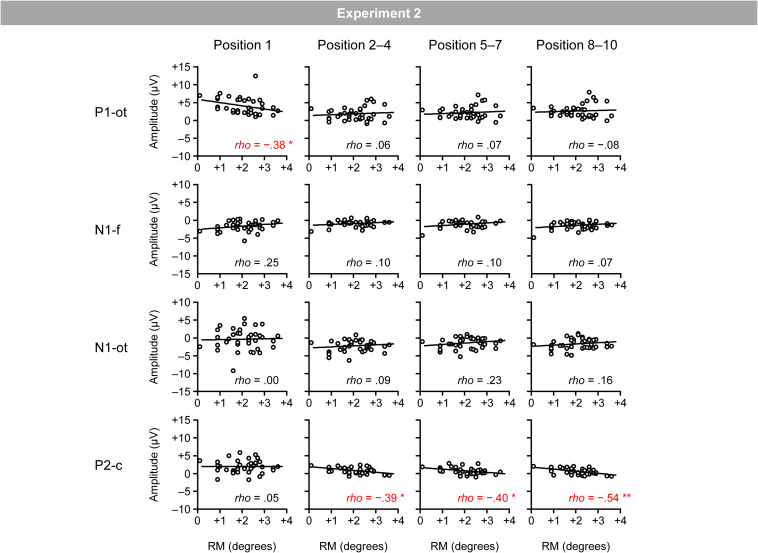
Scatter plots of the relationships between the magnitudes of RM and VEPs. P1-ot: occipito-temporal P1, N1-f: frontal N1, N1-ot: occipito-temporal N1, P2-c: central P2. The linear regression fits to the data are shown. *Indicates *p* < 0.05 and **indicates *p* < 0.01 by Spearman’s correlation analysis (two-tailed).

Figure 8 shows the relationship between the magnitudes of RM and VEPs. Spearman’s correlation analysis (two-tailed) revealed significant negative correlations between the magnitudes of RM and central P2 for the second–fourth (ρ = −0.39; *p* < 0.05), fifth–seventh (ρ = −0.40; *p* < 0.05), and eighth–tenth positions (ρ = −0.54; *p* < 0.01), as well as a significant negative correlation between the magnitudes of RM and occipito-temporal P1 for the first position (ρ = −0.38; *p* < 0.05).^4^

### Discussion

As in Experiment 1, RM robustly occurred in the regular trials, and the inducing stimuli elicited occipito-temporal P1, frontal N1, occipito-temporal N1, and central P2. The magnitude of RM was again negatively correlated with the magnitude of central P2; participants who showed a smaller P2 tended to exhibit greater RM. Thus, the negative correlation between the magnitudes of RM and central P2 observed in Experiment 1 was clearly replicated in Experiment 2, ensuring the replicability and robustness of the negative correlation between RM and central P2. In addition to central P2, the magnitude of occipito-temporal P1 for the first position was negatively correlated with the magnitude of RM. However, given that such negative correlation was not observed in Experiment 1 (rather, a tendency of an opposite, positive correlation was observed in Experiment 1), no conclusion could be drawn about this effect.

## General Discussion

In Experiments 1 and 2, the results regarding the same/different judgment showed that RM clearly occurred in regular trials. This is highly consistent with the previous findings with the conventional RM paradigm with regular rotations of a bar (Freyd and Finke, 1984, 1985) as well as with other types of changes (Kelly and Freyd, 1987; Hayes and Freyd, 2002). The magnitude of RM in Experiment 2 (mean of 2.06°) was numerically greater than that in Experiment 1 (mean of 1.86°). This could be mainly attributed to the step size of a regular rotations of a bar (i.e., 18° in Experiment 1 and 20° in Experiment 2), since the magnitude of RM is proportional to the implied velocity of regular rotations of a bar (Freyd and Finke, 1985; Finke et al., 1986).

In Experiments 1 and 2, the magnitude of RM was negatively correlated with the magnitude of central P2 at around 200 ms after bar onset; that is, participants who showed a smaller P2 tended to exhibit greater RM. This is consistent with the expectation based on a previous finding that the suppression of central P2 is a neural effect that specifically emerges when the current position of a visual object successfully matches the predicted position of the object based on sequential regularities (Kimura and Takeda, 2015). The negative correlations between the magnitudes of RM and central P2 showed a similar time course in Experiments 1 and 2. That is, the magnitudes of RM and central P2 were not initially correlated at the first position, and they started to be negatively correlated at later positions. These results support the idea that the correlation would be associated with the individual’s ability to automatically form a prediction based on sequential regularities and contradict the idea that the correlation between the magnitudes of RM and P2 merely reflects the individual’s inherent strength of neural activations represented by P2.

Although the negative correlations between the magnitudes of RM and central P2 were highly similar between Experiments 1 and 2, there were slight differences between Experiments 1 and 2. The negative correlation appeared earlier in Experiment 2 (i.e., the second–fourth positions) than in Experiment 1 (i.e., the fifth–seventh positions). Also, the negative correlation between the magnitudes of RM and central P2 was more robust (at least in terms of the correlation coefficient) in Experiment 2 than in Experiment 1. These differences would be mainly attributed to two differences in the experimental design. First, they may be attributed to the greater step size of the regular rotation of a bar in Experiment 2 (i.e., 20°) than in Experiment 1 (i.e., 18°). Second, they may be attributed to the arrangement of directions of regular rotation. In Experiment 1, directions of regular rotation (i.e., clockwise and counterclockwise) were changed trial-by-trial in a random manner; therefore, only after the second inducing stimulus was presented, the participants could recognize whether the current regular rotation was clockwise or counterclockwise and could predict the orientation of the upcoming inducing stimuli. In contrast, in Experiment 2, the direction of regular rotation (i.e., clockwise or counterclockwise) was fixed for each participant throughout the experiment; therefore, immediately after the first inducing stimulus was presented, the participants could predict the orientation of the upcoming inducing stimuli.

It appears difficult to attribute the negative correlation between RM and central P2 to factors other than prediction. For example, one might consider that the negative correlation may be involved in visual attention to inducing stimuli. However, if the negative correlation was involved in the degree of visual attention, then significant correlations should have also been observed between RM and occipito-temporal P1/N1, since visual attention predominantly affects occipito-temporal P1/N1 (Hillyard and Anllo-Vento, 1998; Luck et al., 2000). The present results of the almost null correlation between RM and P1/N1 are incongruent with this expectation. One might also consider that the negative correlation may be associated with some strategic processes. It has been shown that RM is primarily determined by automatic predictive processes. However, due to the essential requirements of the task (i.e., the same/different judgment), RM may not be free from the effects of strategic processes such as “cognitive resistance” (i.e., to intentionally stop the forward displacement of a sensory representation to improve the same/different judgment; Finke et al., 1986) and “opposite-acting compensation” (i.e., to strategically change the judgment to compensate for a likely perceptual bias; Joordens et al., 2004). Although such effects of strategic processes could not be completely ruled out, given that the present negative correlation was not limited to the eighth–tenth positions where such strategic processes are expected to be operated, it seems unlikely that the presented negative correlation was related to such strategic processes.

Taken together, the present results suggest that the greater sensory suppression as indicated by smaller central P2 underlies stronger predictive modulation of visual perception as indicated by greater RM. Given the previous findings that neural sources of central P2 were localized around lower visual areas around the striate and prestriate cortices (Capilla et al., 2016; see also Metha et al., 2000) and P2 may be a sign of delayed reactivation of lower visual areas via reentrant feedback projections from higher areas (Di Russo et al., 2003, 2008; see also Olson et al., 2001; Noesselt et al., 2002), the present results would support the notion that the strength of prediction suppression of delayed reactivation of lower visual areas determines the strength of predictive modulation of visual perception. This notion is consistent with that in a human neuroimaging study which demonstrated that successful matching between current visual inputs and predicted visual inputs based on sequential regularities drives less neural activation in the striate cortex, probably via feedback projections from higher visual areas (Alink et al., 2010). From a broader perspective, the present findings appear to be in line with previous findings that the strength of delayed reactivation of lower visual areas such as striate cortex via reentrant feedback projections critically determines perceptual experience and awareness (Lamme et al., 1998; Lamme and Roelfsema, 2000; Tong, 2003; Pak et al., 2020) as well as the hierarchical predictive coding framework which proposes that prior expectations about an upcoming stimulus act as top-down signals that predict the bottom-up input (Rao and Ballard, 1999; Friston, 2005; Summerfield and de Lange, 2014).

The present findings should be treated with caution in three respects. First, the present study used a simple stimulus (i.e., a bar) that changed along with a simple regularity (i.e., rotation). It seems possible that, when observing a more complex stimulus (e.g., face) that changes along with a more complex regularity (e.g., changes in facial features), the main loci of prediction suppression might change (e.g., from lower visual areas to higher areas such as face-responsible inferior temporal cortex, Haxby et al., 2000), and the prediction suppression in such higher areas may mainly determine the predictive modulation of visual perception. Second, the present study applied a conventional RM paradigm (Freyd and Finke, 1984, 1985), but there are several different RM paradigms such as those with a still photograph of an object in motion (Freyd, 1983) or a smooth animated motion of an object (Hubbard and Bharucha, 1988). To capture the overall picture of the relationship between prediction suppression based on sequential regularities and predictive modulation of visual perception, the accumulation of studies with a variety of paradigm should be required. Third, the present study did not directly examine the neural sources of central P2. The precise source localization was not a realistic option in the present study, since central P2 was expected to be overlapped by temporally and/or spatially adjacent VEPs. Furthermore, although a previous study reported that the neural sources of central P2 were localized in lower visual areas (Capilla et al., 2016), stimuli used in the previous study (i.e., reversal of a checkerboard pattern) were different from those used in the present study (i.e., discrete presentation of a bar). In future studies, the direct examination of the neural sources should be made with an optimal experimental design by which the predictive suppression of central P2 can be isolated from other neural activities (Kimura and Takeda, 2015).

Finally, this present finding may drive the fundamental question of what factors determine the individual’s ability to automatically form a prediction based on sequential regularities. For example, previous RM studies showed that the magnitude of RM can be modulated by domain-specific expertise (e.g., greater RM for road scenes in experienced compared to inexperienced automobile drivers), suggesting that prediction ability can be improved with expertise (Blättler et al., 2010, 2011). As another approach, a recent study sought clinical factors that determine the magnitude of RM in terms of autistic and schizotypal traits, although a strong factor could not be determined (Tulver et al., 2019). The quest for critical factors that determine an individual’s prediction abilities would be important for better understanding the mechanisms of visual perception and for establishing possible training/intervention methods to improve prediction abilities.

## Conclusion

By measuring VEPs with a conventional RM paradigm, the present study demonstrated the relationship between the strength of predictive modulation of visual perception (as measured by the magnitude of RM) and the strength of prediction suppression of sensory response (as measured by the magnitude of central P2, which is best assumed to represent delayed reactivation of lower visual areas around striate and prestriate cortices via reentrant feedback projections from higher areas).

## Data Availability Statement

The raw data supporting the conclusion of this article will be made available by the authors, without undue reservation.

## Ethics Statement

The studies involving human participants were reviewed and approved by the Safety and Ethics Committee of the National Institute of Advanced Industrial Science and Technology (AIST). The patients/participants provided their written informed consent to participate in this study.

## Author Contributions

The author confirms being the sole contributor of this work and has approved it for publication.

## Conflict of Interest

The author declares that the research was conducted in the absence of any commercial or financial relationships that could be construed as a potential conflict of interest.

## Publisher’s Note

All claims expressed in this article are solely those of the authors and do not necessarily represent those of their affiliated organizations, or those of the publisher, the editors and the reviewers. Any product that may be evaluated in this article, or claim that may be made by its manufacturer, is not guaranteed or endorsed by the publisher.

## Footnotes

1 In general, VEPs time-locked to visual stimulus onset are comprised of (1) occipital C1 that peaks at around 60 ms, (2) occipito-temporal P1 that peaks at around 110 ms, (3) frontal N1 that peaks at around 140 ms, (4) occipito-temporal N1 that peaks at around 170 ms, and (5) central P2 that peaks at around 200 ms after stimulus onset (Clark et al., 1995; Di Russo et al., 2002; Capilla et al., 2016). The main neural sources of these VEPs were localized in the visual areas and the related areas belonging to the dorsal and ventral processing streams: (1) C1 in the striate cortex (i.e., V1), (2) P1 in the dorsal and ventral extrastriate cortices (e.g., V3 and V4), (3) frontal N1 in the parieto-occipital cortex, (4) occipito-temporal N1 in the dorsal extrastriate cortex (e.g., V3), and (5) P2 in the striate and prestriate cortices (i.e., V1 and V2) (Clark et al., 1995; Di Russo et al., 2002, 2003, 2008; Capilla et al., 2016). Although tentative, occipital C1, occipito-temporal P1, and frontal N1 may mainly represent early bottom-up activation, whereas occipito-temporal N1 and central P2 may mainly represent delayed reactivation of visual areas via reentrant feedback projections from higher areas (Di Russo et al., 2003, 2008; Capilla et al., 2016; for related findings, see Olson et al., 2001; Noesselt et al., 2002). Note that occipital C1 was not analyzed in the present study, since centrally presented visual stimuli (see Figures 1, 2) were not suitable to observe C1; retinotopically specific single-quadrant stimulation is required to observe C1 (Clark et al., 1995).

2 In a certain participant, if the mean percentages of “same” response in the backward 8°, backward 6°, backward 4°, backward 2°, same, forward 2°, forward 4°, forward 6°, and forward 8°conditions were 0, 9, 12, 53, 87, 90, 78, 45, and 12%, respectively, then the sum of the products of the percentage of “same” responses and the distance of the probe from true-same was calculated (i.e., 0^∗^(−8) + 9^∗^(−6) + 12^∗^(−4) + 53^∗^(−2) + 87^∗^(0) + 90^∗^(+2) + 78^∗^(+4) + 45^∗^ (+6) + 12^∗^(+8) = 650), and the obtained value (i.e., 650) was divided by the sum of the percentages of the same responses (i.e., 0 + 9 + 12 + 53 + 87 + 90 + 78 + 45 + 12 = 386). This resulted in the magnitude of RM of 1.68°.

3 Given that VEPs for the first position showed a large and sustained occipito-temporal positivity at around 200–400 ms (see Figures 4A,B), one may be interested in the relationship between RM and the occipito-temporal positivity. So, an exploratory analysis was made for the correlation between the magnitudes of RM and the occipito-temporal positivity (mean amplitudes were calculated with the time window of 240–280 ms at PO8 electrode). The results showed that the magnitude of the positivity was not significantly correlated with the magnitude of RM; the first (*rho* = 0.06, *p* = 0.74), second–forth (*rho* = 0.10, *p* = 0.57), fifth–seventh (*rho* = 0.00, *p* = 0.99), and eighth–tenth positions (*rho* = −0.06, *p* = 0.72).

4 As in Experiment 1, an exploratory analysis for the correlation between the magnitudes of RM and the occipito-temporal positivity (mean amplitudes were calculated with the time window of 240–280 ms at PO8 electrode) showed that the magnitude of the positivity was not significantly correlated with the magnitude of RM; the first (*rho* = 0.05, *p* = 0.76), second–forth (*rho* = 0.13, *p* = 0.46), fifth–seventh (*rho* = 0.16, *p* = 0.34), and eighth–tenth positions (*rho* = 0.03, *p* = 0.87).
